# Cognition, Utilization and Industrial Development of Sports Nutrition Foods: An Evidence-Based Narrative Review

**DOI:** 10.3390/nu18121924

**Published:** 2026-06-13

**Authors:** Yingqi Yao, Lin Zhu

**Affiliations:** 1 School of Health Sciences and Physical Education, Macao Polytechnic University, Macao SAR 999078, China; p2525399@mpu.edu.mo; 2 Laboratory of Exercise Physiology, Guangzhou Sport University, Guangzhou 510075, China; 3 Innovative Research Center for Sports Science in the Guangdong-Hong Kong-Macao Greater Bay Area, Guangzhou Sport University, Guangzhou 510500, China

**Keywords:** national fitness, sports nutrition foods, cognition level, nutrition strategy, food development

## Abstract

**Background/Objectives:** To clarify the cognitive level and selection behavior of the exercising population regarding sports nutrition foods, as well as their relationship with athletic performance, this narrative review examined the literature published from 2001 to 2025. **Methods:** CNKI, Wanfang Data, PubMed, and ScienceDirect were searched for studies published between 2001 and 2025 using keywords: sports nutrition foods, exercise intensity, public cognition, and food development. Studies addressing ingredient functionality, exercise-related nutritional requirements, public cognition, or product development were included. After screening, 45 full-text articles and four authoritative documents were incorporated into the synthesis. **Results:** The synthesis reveals a persistent disconnect between the cognition and utilization of sports nutrition foods. Common misconceptions include inappropriate supplementation timing, indiscriminate product selection, and imprecise dosage control, while structural constraints on the industrial side—product homogenization, inadequate standardization, and imprecise product development—remain significant barriers. **Conclusions:** To bridge this gap, we recommend establishing a three-in-one public education framework that integrates professional education, mass media communication, and regulatory oversight, and we encourage enterprises to transition toward clean labeling, precision nutrition, and green processing. This review provides an evidence-based reference for advancing the development of sports nutrition foods.

## 1. Introduction

As national fitness has become a national strategic priority, a science-based understanding and appropriate use of sports nutrition foods have become pivotal to public health. Data from the Sixth National Physical Fitness Survey show that while the overall pass rate reached 84.9%, the excellence rate was only 30.9%. This gap points to a structural disconnect: participation in national fitness has reached a considerable scale in terms of quantity, yet substantial room remains for improvement in quality. The contribution of physical training relative to proper nutrition in achieving optimal fitness outcomes has been estimated at approximately 1:9 [1], underscoring the central role of nutritional intervention in optimizing athletic performance.

However, the application and development of sports nutrition foods face numerous challenges. On the demand side, the general public commonly holds cognitive misconceptions regarding supplementation timing, product category selection, and dosage control [2]. On the supply side, the industry faces structural constraints, including product homogenization, inadequate standardization, and insufficient precision in product development. These issues can be examined from three perspectives.

At the mechanistic level, research has primarily validated the physiological functions of core nutritional ingredients. Studies on proteins and amino acids have covered key components such as whey protein [3], branched-chain amino acids [4], and glutamine [5]. Among carbohydrates and adjunctive ingredients, the mechanisms of creatine [6] and vitamins [7] are relatively well established. As an emerging raw material, plant proteins have been the subject of 106 patents and 35 publications over the past decade [8].

At the application level, significant differences in nutritional requirements among diverse populations have been revealed. Adolescent athletes require consideration of both energy supply and skeletal development [9,10,11,12]; older adults face the coexistence of malnutrition and excess energy intake, with sarcopenia intervention being particularly critical [13,14,15,16]; and female athletes need distinct macronutrient recommendations [17,18]. Consumption behaviors among non-athletes exhibit a combination of preference for protein products and biased risk perception [19].

At the industrial level, research has focused on the establishment of sports food standards and market development. The current standard framework is based on GB 24154-2015 [20], further refined by T/CEAC 009-2024 [21], but considerable scope for improvement remains [22]. According to an industry analysis, the market scale reached RMB 9.71 billion in 2024 and is projected to rise to RMB 20.93 billion by 2030 [23]; these figures, given their commercial origin, should be interpreted with appropriate caution. This rapid expansion underscores the urgent need for precision-oriented development [24,25,26].

A review of the extant literature reveals two prominent characteristics. First, research perspectives tend to be relatively narrow, mostly concentrating on the mechanisms of single ingredients or the needs of specific populations, and lacking an integrative analysis that incorporates exercise intensity, metabolic characteristics, and nutritional strategies into a unified framework. Second, industry-related studies remain largely descriptive and seldom construct precision development pathways grounded in the heterogeneity of population needs. Accordingly, this paper attempts to construct an integrative analytical framework of “intensity classification–metabolic analysis–strategy matching–industrial reflection.” It first examines the differential nutritional strategies across exercise scenarios based on the metabolic and energy demands at varying exercise intensities [6,9,27,28,29,30,31,32], then conducts an in-depth analysis of the specific needs of particular populations [9,11,12,13,14,15,16,18,33], and finally extends to the design and development directions for sports nutrition foods [8,20,21,22,24,26], with the aim of providing an evidence-based reference for health promotion and industrial upgrading.

## 2. Scope and Methods

### 2.1. Definition of the Research Object

For the purpose of this paper, sports nutrition foods refer specifically to food categories designed or selected to meet the needs of exercising individuals in terms of specific physiological metabolic states, athletic performance enhancement, and special nutrient supplementation. According to the definition in GB 24154-2015 [20], sports nutrition foods are “foods specially processed to meet the physiological metabolic states, exercise capacity, and special needs for certain nutrients of the exercising population (referring to individuals who participate in physical exercise at least 3 times per week, for at least 30 min each time, and at an intensity of moderate or above).” Based on a systematic review of relevant food standards [34], the functional scope of sports nutrition foods mainly covers the three temporal phases before, during, and after exercise. The core functional ingredients and their corresponding physiological effects can be summarized as follows:

#### 2.1.1. Proteins and Amino Acids

This category includes proteins derived from plants and animals, whey protein, casein, and others. Wang et al. systematically reviewed the functional characteristics of whey protein, showing that timely supplementation of high-quality protein after exercise is a critical step in promoting skeletal muscle repair and remodeling [3]. Current metabolic estimates suggest a recommended daily protein intake of approximately 60–80 g for adults. Studies at the amino acid level have confirmed that supplementation with branched-chain amino acids and glutamine provides well-documented benefits [4,5]. Notably, plant proteins are increasingly used as novel raw materials for sports nutrition foods. A review by Kostrakiewicz-Gierałt, published in Nutrients, systematically summarized the research progress and application potential of plant-derived proteins in sports nutrition, supporting the exploration of innovative raw material development in this field [8]. Furthermore, Jahan-Mihan et al. compared the effects of whey, casein, soy, pea, and mixed proteins across different training modalities [31], while Nederveen et al. focused on the elderly population, discussing the evidence base for protein supplementation in the management of sarcopenia [16].

#### 2.1.2. Minerals

Minerals are involved in the fine regulation of body fluid osmotic pressure, acid–base balance, and neuromuscular excitability. Substantial sweating during exercise can lead to the loss of electrolytes such as sodium, potassium, calcium, and magnesium [35], which cannot be overlooked in the exercising population. At the skeletal level, the synergistic effect of protein and minerals is equally critical, exerting a direct influence on athletic performance and injury prevention [33].

#### 2.1.3. Vitamins

Vitamins act as coenzymes in the regulation of energy metabolism. Research has confirmed that vitamins have a clear mitigating effect on exercise-induced fatigue—supplementation with vitamin A and B vitamins can effectively delay the onset of fatigue, while the antioxidant system composed of vitamins A, C, and E can efficiently scavenge the free radicals generated in abundance after high-intensity exercise [7].

#### 2.1.4. Carbohydrates

Carbohydrates, including monosaccharides, disaccharides, and polysaccharides, represent the most direct and efficient energy substrate during exercise. The co-administration of creatine with carbohydrates exhibits a synergistic effect in enhancing explosive power. A position stand of the International Society of Sports Nutrition indicates that creatine supplementation has well-defined efficacy and a favorable safety profile in improving high-intensity exercise performance [6]. A systematic review by Dos Santos et al. further confirmed that carbohydrate supplementation positively influences endurance performance and capacity, facilitating glycogen store recovery between training sessions [32]. A systematic review of 46 studies by Yu and Ding also demonstrated that dietary supplements such as caffeine and β-alanine improve athletic performance to varying degrees in elite athletes [36].

#### 2.1.5. Lipids

The Dietary Guidelines for Chinese Residents (2022) recommend that the proportion of energy derived from dietary fat should not exceed 30% of total energy intake and emphasize a balanced consumption of saturated, monounsaturated, and polyunsaturated fatty acids [27]. Adequate fat intake also contributes to maintaining normal hormone levels and the absorption of fat-soluble vitamins; excessive fat restriction may be detrimental to long-term exercise adaptation and bodily function maintenance. Additionally, the gut microbiota can participate in fat metabolism and lactate degradation, playing a positive role in modulating exercise-induced fatigue and maintaining metabolic homeostasis.

### 2.2. Literature Search and Study Selection

This review was conducted as an evidence-based narrative review. The literature was identified through a multi-pronged approach combining systematic database searches, backward citation tracking, and targeted retrieval of authoritative documents.

Database searches: Four electronic databases—China National Knowledge Infrastructure (CNKI), Wanfang Data, PubMed, and ScienceDirect—were systematically searched from January 2001 to March 2025. The search strategy was structured around four thematic blocks, using the Boolean operator AND between blocks OR within each block:

Block 1—Sports nutrition foods: “sports nutrition food” OR “sports nutrition supplement” OR “sports nutrition product” OR “exercise nutrition food” OR “sports drink” OR “protein supplement” OR “dietary supplement”.

Block 2—Exercise and fitness context: “exercise” OR “sport” OR “athlete” OR “fitness” OR “physical activity” OR “training” OR “national fitness” OR “exercise intensity”.

Block 3—Cognition and utilization: “cognition” OR “knowledge” OR “attitude” OR “perception” OR “awareness” OR “behavior” OR “consumption” OR “utilization” OR “use”.

Block 4—Strategy and development: “nutrition strategy” OR “supplementation strategy” OR “nutritional intervention” OR “food development” OR “product design” OR “industrial development” OR “standard”.

In CNKI and PubMed, the complete Boolean expressions were executed as single queries, yielding 40 and 32 records, respectively. In ScienceDirect, the search was conducted using the field code title-abs-key. To comply with the platform-imposed limit of eight Boolean connectors per search field, the search was split into three consecutive queries: Block 1 paired with Block 2 yielded 18 records; Block 1 with Block 3, 5 records; and Block 1 with Block 4, 2 records. After deduplication, 19 unique records were retained. In Wanfang Data, manual combinations of the keywords were applied, yielding 21 records. The complete search expressions for each database are provided in Supplementary Table S1. After deduplication across databases, a total of 112 unique records were obtained for title and abstract screening.

Supplementary identification: The database searches seeded the review and defined its core thematic scope. Most included articles were then identified through backward citation tracking of the retrieved articles and key reviews, supplemented by targeted searches informed by the authors’ knowledge of the field. Four authoritative documents—two national/industry standards (GB 24154-2015 and T/CEAC 009-2024), one dietary guideline (Chinese Nutrition Society, 2022 [27]), and one industry market report—were retrieved through targeted searches of official websites and databases.

Study selection and eligibility criteria: Studies were included if they met all of the following criteria: (a) they addressed the functional properties of sports nutrition food ingredients; (b) they examined nutritional requirements under different exercise loads; (c) they investigated the cognition, attitudes, or utilization behaviors of sports nutrition foods among exercising populations; or (d) they discussed the design, development, or standardization of sports nutrition food products. Eligible study designs comprised original research (cross-sectional studies, cohort studies, randomized controlled trials), systematic reviews, meta-analyses, narrative reviews, and authoritative official documents (national/industry standards, dietary guidelines). Studies were excluded if they: (a) focused exclusively on clinical populations without reference to exercise or sports contexts; (b) discussed general dietary patterns without specific reference to sports nutrition foods; (c) were conference abstracts, editorials, or commentaries lacking primary or synthesized data; or (d) were duplicate records.

Screening process: One reviewer screened titles, abstracts, and full texts, and a second reviewer verified all inclusion and exclusion decisions. Disagreements were resolved through discussion. During title and abstract screening, records clearly falling outside all four inclusion criteria—such as general nutrition studies or clinical nutrition unrelated to sports—were excluded.

Synthesis: After full-text screening, 45 articles met the inclusion criteria. Together with the four supplementary authoritative documents, a total of 49 references were included in the final review. The included literature was thematically synthesized around three core areas: (a) energy metabolism and nutritional demands across different exercise intensities; (b) scenario-based sports nutrition strategies; and (c) targeted product design and industrial development.

## 3. Results and Analysis

### 3.1. Physiological Metabolism and Energy Demands of Physical Exercise

The scientific basis of sports nutrition is rooted in the precise classification of exercise load intensity and a profound understanding of its corresponding metabolic characteristics. A study explored the relationship between energy expenditure and energy replenishment by analyzing the movement characteristics and energy metabolism of various sports [29]. A clear logical mapping exists among exercise intensity, metabolic features, and nutritional requirements (Table 1).

#### 3.1.1. Exercise Intensity Classification and Metabolic Characteristics

The intensity thresholds adopted in this review follow the general classification framework of the American College of Sports Medicine, which defines light intensity as <40% of heart rate reserve or <120 beats/min, moderate as 40–59% (120–150 beats/min), and vigorous as ≥60% (>150 beats/min). The corresponding MET values align with the Compendium of Physical Activities and the Dietary Guidelines for Chinese Residents (2022) [27].

Following the ACSM classification framework described above, exercise intensity can be divided into three levels: low, moderate, and high.

Low-intensity exercise is primarily supported by aerobic metabolism, with the substrate mobilization sequence being carbohydrate, fat, and protein. This type of exercise can be sustained for a long duration, imposes a relatively small load on the body and is suitable for individuals with low baseline fitness and the elderly for maintaining basic cardiorespiratory function.

During moderate-intensity exercise, the body relies on a mixed supply of aerobic and anaerobic metabolism. After approximately 30 min of exercise, the proportion of fat mobilized increases significantly, becoming a major fuel source. For adolescent athletes, sports training before the age of 12 should predominantly consist of this intensity, focusing on the development of aerobic endurance and motor skill acquisition.

High- and very-high-intensity exercise is dominated by anaerobic glycolysis. The pre-existing ATP-CP system can only sustain maximal output for approximately 6–8 s (e.g., a 100 m sprint), after which energy is rapidly supplied primarily through the anaerobic breakdown of glycogen, accompanied by the accumulation of metabolic by-products such as lactate and the onset of fatigue.

#### 3.1.2. Energy Expenditure Estimation and Principles of Replenishment

The metabolic equivalents (METs) vary significantly across different exercise intensities. Using the basal metabolic rate calculation formulas and activity coefficients recommended by the Chinese Nutrition Society (Table 1), an individual’s estimated daily caloric requirement can be derived [27]. The MET values for low-intensity exercise range from 1.6 to 2.9, with relatively limited energy expenditure. In contrast, energy expenditure per unit of time increases markedly during moderate-intensity (MET 3–6) and high-intensity (MET > 6) exercise. Therefore, when formulating nutritional supplementation plans, the energy contribution ratio of macronutrients must be dynamically adjusted based strictly on exercise duration and intensity level.

### 3.2. Selection Strategies and Scenario-Based Application of Sports Nutrition Foods

Appropriate nutrient supplementation supports athletic performance while strengthening muscles and bones, thereby reducing the risk of sports injuries. Carbohydrates are the body’s primary energy substrate. Glycogen storage capacity correlates positively with the duration of prolonged aerobic training. Reasonable fat intake does not impose an excessive burden on the body. The core of sports nutrition supplementation lies in the precise targeting of critical time windows. Naderi et al. reviewed post-exercise recovery nutritional strategies, covering carbohydrate intake for glycogen replenishment, as well as the impact of protein type and dosage on muscle recovery and nitrogen balance [30].

#### 3.2.1. Precision Supplementation in Moderate-to-High-Intensity Training and Competition Scenarios

For professional athletes and advanced fitness enthusiasts, the timing of nutrient supplementation needs to be precise down to the minute.

**Pre-Training/Pre-Competition Phase (2–4 h):** The core focus is glycogen loading and appropriate hydration. If creatine supplementation is desired, it is recommended to be ingested 30 min before training to enhance explosive performance [6]. The optimal timing of creatine intake, however, remains unsettled. Although pre-exercise ingestion is widely practiced, the ISSN position emphasizes that total daily creatine saturation—rather than acute timing relative to exercise—is the primary determinant of ergogenic benefit [6]. Precise timing recommendations should therefore be regarded as provisional rather than definitive.

**During Training/Competition (Intermittent Periods):** The core aim is to maintain blood glucose stability and electrolyte balance. A systematic review by Dos Santos et al. indicated that carbohydrate intake facilitates glycogen store recovery between training bouts, helping to sustain exercise intensity and performance. Sports drinks, however, are not suitable for everyone due to their formulation [37].

**Post-Training/Post-Competition Phase:** It is suggested that 30–40 min after exercise, priority may be given to consuming alkaline-rich foods such as fresh vegetables and fruits, followed by regular intake of high-quality protein [3]. For this phase, creatine supplementation is recommended within 2 h post-exercise, co-ingested with carbohydrates, to accelerate glycogen resynthesis and muscle repair [6]. Although the concept of alkaline foods for post-exercise recovery appears in several sports nutrition texts, direct evidence linking dietary alkalinity to accelerated recovery remains limited, and this recommendation should be viewed as a prudent practice suggestion rather than a firmly established strategy. Glutamine supplementation may attenuate certain fatigue markers [5]. Additionally, a review by Jahan-Mihan et al. further noted that protein dosage and timing strategies should be adjusted according to training type, with older athletes potentially requiring a higher single dose to counteract anabolic resistance [31]. Notably, the ISSN has stated that protein supplementation need not strictly adhere to a 30–60 min post-exercise window and that distributing intake evenly throughout the day may be more critical for muscle protein synthesis [38]. While this position is supported by multiple studies, the practical emphasis on post-exercise protein intake within a short time window remains common in applied guidelines. This discrepancy highlights the need for further research reconciling laboratory findings with field-based recommendations, which could open new optimization avenues for competitive athletes aiming to improve athletic performance.

#### 3.2.2. Nutritional Focus for the General Fitness Population

The vast majority of sports and exercise participants are individuals pursuing a healthy lifestyle, and the application scope of sports nutrition foods has gradually expanded from elite athletes to the broader general fitness population [28]. A cross-sectional study by Chapple et al., published in Nutrients, surveyed 307 non-athlete adults in Australia and confirmed that protein products dominated consumption, with protein powder at 82% and protein bars at 61%. Supermarkets remained the primary purchasing channel (52%), while mass media served as the leading source of product recommendations (39%). These findings clearly reflect the typical consumption motives and biased risk perceptions among ordinary exercisers [19].

For resistance training practitioners aiming for muscle hypertrophy, it is necessary to maintain a positive post-training nitrogen balance through adequate high-quality protein intake to promote muscle repair and synthesis [38]. For individuals targeting fat loss, a training sequence of “resistance exercise first, then aerobic exercise” is advisable and nutritional foods should preferentially be of the high-dietary-fiber, low-energy-density type to maintain satiety and muscle mass while controlling caloric intake.

### 3.3. Utilization and Targeted Design of Sports Nutrition Foods

#### 3.3.1. Nutritional Interventions to Alleviate Exercise-Induced Fatigue

The causes of exercise-induced fatigue involve three dimensions: depletion of energy substrates, accumulation of metabolic by-products, and disturbance of internal environment homeostasis. Nutritional intervention should be integrated throughout the entire pre-, during, and post-exercise process; the following analysis focuses on post-exercise recovery strategies. Taking “energy substrate depletion” as an example, sustained moderate-to-high-intensity exercise can lead to significant muscle glycogen depletion within approximately 30 min, directly affecting the maintenance of athletic performance [10] and subsequently influencing competitive results. In response to these mechanisms, several nutritional strategies can be adopted. Post-exercise antioxidant supplementation helps remove excess free radicals [7], and specific branched-chain amino acids can inhibit muscle protein breakdown [4,5]. Dietary patterns that emphasize water and electrolyte replenishment, along with alkaline foods, may accelerate fatigue recovery [39]. Anti-fatigue functional ingredients naturally present in foods can also be utilized [40]. A systematic review of 46 studies by Yu and Ding further assessed the efficacy of dietary supplements in elite athletes, finding that pre-exercise caffeine can enhance performance and improve technical movement accuracy and stability, while amino acids and probiotics show potential for fatigue prevention and exercise tolerance [36].

#### 3.3.2. Nutritional Support for Bone Health and Muscle Loss Prevention

Bone development, maintenance, and repair all depend on an adequate supply of calcium. Protein is an important nutrient for maintaining bone health in addition to calcium and vitamin D [33], as previously defined. For the middle-aged and elderly population, as well as adolescent athletes, the combined supplementation of calcium and collagen has a synergistic effect. Physical exercise and nutritional supplements play an interventional role in age-related sarcopenia [14]. The combination of compromised nutritional status and excessive caloric intake in older adults means that product design must reconcile high nutrient density with an appropriate texture [13]. A systematic review and meta-analysis by Xie et al. found that resistance training combined with amino acid supplementation improved muscle strength and physical function in older adults with sarcopenia [15]. Nederveen et al. reviewed multi-ingredient supplements for sarcopenia and sarcopenic obesity and found that resistance training combined with protein supplementation—particularly whey and casein—benefits older adults, with a protein intake ceiling of 1.5–1.7 g/kg/day [16]. For adolescent athletes, the value of collagen supplementation lies primarily in the prevention of sports injuries. High-intensity specialized training imposes sustained stress on tendons, ligaments, and articular cartilage; collagen supplementation (especially when co-ingested with vitamin C) can stimulate collagen synthesis in connective tissues, enhancing the tensile strength of tendons and ligaments and thereby reducing the risk of stress-induced injuries.

#### 3.3.3. Design and Development Pathways for the Sports Nutrition Food Industry

The following design directions are based on a synthesis of the reviewed literature, industry standards, and market analyses. The strength of supporting evidence varies, and recommendations grounded primarily in market reports or narrative reviews should be regarded as forward-looking rather than definitive. Currently, sports nutrition foods are mostly categorized and designed based on function. From a market supply perspective, three structural constraints are evident in this sector. First, the degree of product differentiation is insufficient. Product design remains at a coarse classification of endurance and strength types, the efficacy evaluation system is not yet sound, and enterprises lack scientific guidance for precision development. Second, the efficiency of processing technology pathways needs improvement. Traditional thermal sterilization processes can cause structural damage to heat-sensitive vitamins and bioactive substances, presenting a technical contradiction between sterilization efficacy and nutrient fidelity. Third, there is an absence of population-specific adaptability in product design, with a structural mismatch between the existing product supply and the physiological characteristics and nutritional needs of specific populations.

Among these structural constraints, the absence of population-specific design is particularly salient. The needs of the following four groups warrant in-depth examination.

**Students and Adolescent Athletes:** This group faces the dual metabolic demands of growth and high-intensity training. Nutritional products must therefore balance energy supply with nutrient preservation and ensure that macronutrient ratios align with these combined demands. In terms of actual consumption, oligosaccharide solid sports beverages are the most frequently used product type in this group [9]; however, dietary supplementation plans for different sports, such as athletics and football, clearly require more refined differentiation [11,12]. At the product formula level, the core is to ensure that the energy contribution ratio of macronutrients aligns with sport-specific requirements—endurance-type sports should emphasize carbohydrate reserves, while strength-type sports should emphasize the intake of high-quality protein blends. These suggestions are largely extrapolated from general sports nutrition principles; sport-specific evidence remains limited [9,11,12].

**Older Adults:** This population exhibits the characteristic coexistence of “malnutrition and energy excess” [13]. Product design must balance high nutrient density with suitable texture. Soft or liquid forms can reduce barriers to oral intake, while the combined supplementation of protein, calcium, and vitamin D should address the risks of muscle loss and bone density decline [14]. Resistance training combined with amino acid and multi-ingredient supplementation provides evidence-based support for these approaches [15,16]. The evidence for protein supplementation in sarcopenia is relatively robust, supported by systematic reviews and meta-analyses [15,16], whereas recommendations for calcium–collagen combinations remain more preliminary [13].

**Weight Management Groups:** Consumer behavior studies among fitness populations [41] and surveys of non-athletes [19] provide empirical references for consumer behavior. Commercially available meal replacement products commonly adopt a formulation strategy of low energy density and high dietary fiber content, but the comprehensiveness and bioavailability of micronutrients are often difficult to guarantee. The core challenge in this field is how to achieve a balanced supply of all nutrients within the constraints of an energy-restricted framework. The behavioral evidence in this group is primarily observational [19,41], and controlled trials are needed to establish optimal supplementation strategies.

**Immune Enhancement Groups:** Nutritional interventions for this population should follow the principle of systematic design. Adhering to the dietary diversity principle outlined in the Dietary Guidelines for Chinese Residents (2022) [27] of “more than 12 food types per day and more than 25 per week,” product design should simultaneously implement strategies for controlling salt, sugar, and oil.

Based on the above analysis, several forward-looking directions for industrial upgrading can be identified. It should be noted, however, that the evidence base for these proposals is more heterogeneous than that for the physiological recommendations discussed in earlier sections. Industrial upgrading should revolve around the two core concepts of “clean labeling” and “green manufacturing.” These structural constraints in the application of sports nutrition foods call for targeted solutions [26]. Specific pathways include: replacing chemical preservatives with the antimicrobial activity of natural plant extracts; extending product shelf life through low-temperature storage and cold chain management; employing vacuum replacement or nitrogen-filling packaging technology to inhibit lipid oxidation; and introducing dynamic sterilization processes that reduce heat-induced nutrient destruction while ensuring microbiological safety. Small and medium-sized food enterprises generally face financial barriers regarding advanced processing equipment; how to lower application costs through regional collaboration or technology sharing is also an unavoidable practical issue in the process of technological implementation [26]. Therefore, the design and development of the sports nutrition food industry must achieve a paradigm shift: from coarse classification to precision nutrition, from single-function to systematic adaptation, and from traditional processes to green manufacturing.

## 4. Discussion

### 4.1. Significant Divergence at the Cognitive Level

Cognitive gaps and inappropriate supplementation behaviors have been widely documented. These represent a major barrier to evidence-based sports nutrition practice.

Adolescent athletes and professional fitness enthusiasts tend to pursue high-purity, single-function supplements, whereas the general public is more susceptible to the influence of commercial advertising. An analysis of social media data revealed significant cognitive biases among consumers regarding sports nutrition [42]. A cross-sectional study of Italian adults found that knowledge of nutraceuticals and dietary supplements was inadequate across all groups [43]. A systematic review of dietary supplement use among gym users indicated substantial heterogeneity in supplementation practices across different fitness levels [44]. Domestic research on Chinese fitness populations has also revealed discrepancies between consumer cognition and behavior [41]. Nutrition consensus guidelines for athletes continue to be updated [45,46,47], and studies of non-athlete populations have identified significant biases in both consumption motives and risk perception related to sports foods [19]. Taking creatine as an example, common misunderstandings about its efficacy, safety, and usage persist widely among both athletes and non-athletes to this day [48,49]. Hence, the popularization of sports nutrition knowledge requires a stratified design.

The mechanisms underlying this low public awareness appear to be threefold. First, the rapid commercialization of sports nutrition products has outpaced the dissemination of evidence-based knowledge, with marketing claims often substituting for scientific guidance. Second, sports nutrition education remains largely absent from public health curricula and primary healthcare settings, leaving consumers reliant on fragmented online sources and peer recommendations. Third, the inherent complexity of exercise metabolism—where optimal supplementation depends on exercise type, intensity, duration, and individual physiological status—makes generic public messaging both difficult to formulate and prone to misinterpretation. Together, these factors form a self-reinforcing cycle in which low health literacy, commercial influence, and the absence of professional guidance perpetuate widespread misconceptions.

These patterns are not unique to China. Studies from Australia [19], Italy [43], and multinational gym-user populations [44] document similarly low levels of sports nutrition literacy and analogous reliance on commercial sources for supplementation guidance, suggesting that the cognitive gap identified in this review reflects a widespread public health challenge rather than a geographically confined phenomenon. Cross-national comparisons further indicate that regulatory frameworks for sports nutrition products vary substantially; some jurisdictions mandate more stringent pre-market evaluation of health claims than others. This disparity may influence both consumer protection and industry behavior.

### 4.2. Standard Construction at the Industry Level

In the domain of sports nutrition foods, the successive promulgation of GB 24154–2015 and T/CEAC 009–2024 [20,21] has established a basic framework for the standards system. However, many gaps remain to be filled in areas such as product standards for niche populations, functional claim evaluation, and detailed market supervision regulations. The incompleteness of the standards system first manifests as a constraint on industrial maturity [22]: from the product side, enterprise R&D lacks precise guidance, trapping it in homogeneous competition [24]; from the application side, the deep integration of sports nutrition foods and sports practice also faces barriers due to standards [26]. Therefore, a critical step in industrial upgrading is the construction of a full-chain standards system that spans raw material traceability, efficacy evaluation, population-specific adaptation, and market supervision.

### 4.3. Channel Construction at the Guidance Level

At present, the majority of the fitness population still lacks stable and professional channels for personalized sports nutrition guidance. To translate sports nutrition knowledge from paper to individual meal plans and training programs, relying solely on fragmented commercial promotion or piecemeal self-education through various media is clearly insufficient. A more effective approach may therefore be to connect communities, schools, and fitness venues into a multi-tiered, accessible nutrition guidance network that functions as a health and wellness service platform. The institutionalized daily one-hour physical activity session in primary and secondary schools could be fully utilized as a natural setting for sports nutrition education. Such integrated efforts may help bridge the gap between “knowing” (cognition) and “doing” (utilization).

### 4.4. Limitations

This review has several limitations that should be acknowledged. First, as a narrative review, the literature selection was not guided by a pre-registered systematic protocol, and the narrative synthesis approach may involve a degree of subjective selection bias inherent to the narrative approach. Second, although four major databases were searched, the review was restricted to publications in Chinese and English, which may have excluded relevant studies published in other languages.

Third, the inclusion criteria were deliberately broad to capture diverse perspectives—encompassing studies on ingredient functionality, population-specific nutritional requirements, public cognition, and product development. This breadth enhances comprehensiveness but inevitably introduces methodological heterogeneity; findings from systematic reviews, cross-sectional surveys, and market reports are synthesized alongside one another, and readers should weigh the certainty of each conclusion according to the strength of its underlying evidence. Where possible, we have indicated the nature of the supporting evidence in the text.

Fourth, the evidence supporting several supplementation strategies comes from studies with considerable protocol variability. Dosage, timing, and ingredient combinations have not yet been standardized across investigations, and study populations are often elite athletes, which may limit the transferability of findings to recreational exercisers. Moreover, as discussed in Section 4.2, the composition and quality of commercially available sports nutrition products can vary substantially, and regulatory oversight of label claims remains incomplete. Translating research evidence into product-specific recommendations therefore warrants caution.

Fifth, several of the population-specific studies cited are based on geographically limited samples that may not fully represent national or international patterns. Finally, the industry-related market data cited for size projections derive from commercial reports rather than peer-reviewed research and should therefore be interpreted with appropriate caution. Despite these limitations, the evidence synthesized here provides a useful starting point for future research and for the design of more targeted sports nutrition strategies.

## 5. Conclusions

Targeting the structural discrepancy revealed by the Sixth National Physical Fitness Survey—wherein the pass rate is 84.9% but the excellence rate is only 30.9%—and the urgency of improving physical fitness, this paper uses sports nutrition foods as an entry point. It constructs an integrative framework of “intensity classification–metabolic analysis–strategy matching–industrial reflection” around the nutritional supplementation strategies of the exercising population in fitness, training, and competition scenarios, and conducts an analysis from the three dimensions of cognition, utilization, and product development and design. The main conclusions are as follows:

First, at the cognitive level, the public’s scientific understanding of sports nutrition foods remains superficial. Inappropriate supplementation timing, indiscriminate product selection, and imprecise dosage control are the prominent current misconceptions. This gap between “knowing” and “doing” directly undermines the contribution of nutritional intervention to athletic performance optimization. Promoting sports nutrition knowledge beyond professional circles is essential. Embedding foundational nutrition education into school physical education and health curricula, and establishing early nutrition screening for young sports talents, would help guide the public from “supplementing by intuition” to “using based on evidence”—a crucial pathway for transitioning the fitness population from “qualified” to “excellent” physical fitness.

Second, at the utilization level, the three temporal phases before, during, and after exercise correspond to distinctly different nutritional needs: the pre-exercise phase focuses on glycogen storage and adequate hydration; the during-exercise phase concerns blood glucose stability and electrolyte balance; the post-exercise phase may benefit from alkaline foods that help buffer metabolic acidity and regular intake of high-quality protein. Differences in energy supply modes across exercise intensities further determine which functional ingredients to select and when to supplement them. Systematic exploration in this field has contributed to the evidence base for sports nutrition. Notably, the International Society of Sports Nutrition indicates that protein supplementation does not require strict adherence to a rigid time window. Future research can further focus on the metabolic characteristics and nutritional requirements of different populations to explore more refined nutritional intervention programs.

Third, at the design level, the dual demands of development and training in adolescent athletes, the concurrent decline of muscle and bone mass in middle-aged and elderly populations, and the differentiated macronutrient requirements of female athletes constitute the underlying logic for precision product development. Consensus statements from the International Society of Sports Nutrition and specialized studies on endurance sports further indicate that the sports nutrition food industry needs a paradigm shift: from coarse classification to precision nutrition, from single-function to systematic adaptation, and from traditional processes to green manufacturing. Green processing technologies—plant-based alternative proteins, clean labeling and shared ultra-high-pressure sterilization—are critical to advancing this industrial transformation and upgrading.

It should be acknowledged, however, that the education–communication–regulation framework proposed in this review faces practical constraints in its implementation. Embedding sports nutrition education into school curricula demands teacher training, dedicated instructional time, and sustained policy support that may not be readily available. Community-based guidance networks require long-term funding and qualified personnel, and the regulatory oversight of health claims on sports nutrition products necessitates both technical expertise and inter-agency coordination. The feasibility of this framework therefore depends on incremental adoption and multi-sector collaboration, rather than any single top-down initiative.

In summary, three developments provide directional guidance for future research and development of sports nutrition foods: the demand feedback generated by improved public cognition, the distinct metabolic characteristics and nutritional needs of different populations, and the ongoing improvement of industrial standards and processing technologies. These developments hold practical significance for advancing the high-quality development of national fitness and for driving the transition in physical fitness from “qualified” to “excellent.”

## Figures and Tables

**Table 1:** Logical mapping between exercise intensity and nutritional requirements.

Dimension	Low Intensity	Moderate Intensity	High/Very High Intensity
Heart Rate	<120 beats/min	120–150 beats/min	>150 beats/min
MET Value	1.6–2.9	3–6	>6
Energy System	Predominantly aerobic	Mixed aerobic/anaerobic	Predominantly anaerobic glycolysis
Fuel Characteristics	Substrate order: carbohydrate → fat → protein	Significant increase in fat mobilization after 30 min	ATP-CP sustains 6–8 s; large accumulation of lactate
Nutritional Strategy	Emphasize nutritional completeness; focus on basic diet; control total calories	Carbohydrate + electrolyte supplementation; regular protein intake; antioxidant intake	Precision supplementation strategies; creatine/BCAA/fast-acting carbohydrates; strictly timed supplementation

Note: MET, metabolic equivalent of task; BCAA, branched-chain amino acids. Heart rate and MET thresholds follow the American College of Sports Medicine classification framework. Nutritional strategy recommendations are synthesized from the evidence reviewed in Section 3.2 and Section 3.3.

## Data Availability

No new data were created or analyzed in this study. Data sharing is not applicable to this article.

## Supplementary Materials

The following supporting information can be downloaded at: https://www.mdpi.com/article/10.3390/nu18121924/s1, Figure S1: Schematic overview of the integrative analytical framework; Table S1: Full search strategies for each database.
