# Supplementary material for: Cognition, Utilization and Industrial Development of Sports Nutrition Foods: An Evidence-Based Narrative Review
Source: Nutrients. 2026 Jun 13;18(12):1924. doi: 10.3390/nu18121924 (PMC13305693; doi:10.3390/nu18121924)
Supplement: Supplementary file 1 [file nutrients-18-01924-s001.zip › Supplementary_Figure_S1.pdf.pdf]

Figure S1: Schematic overview of the integrative analytical framework

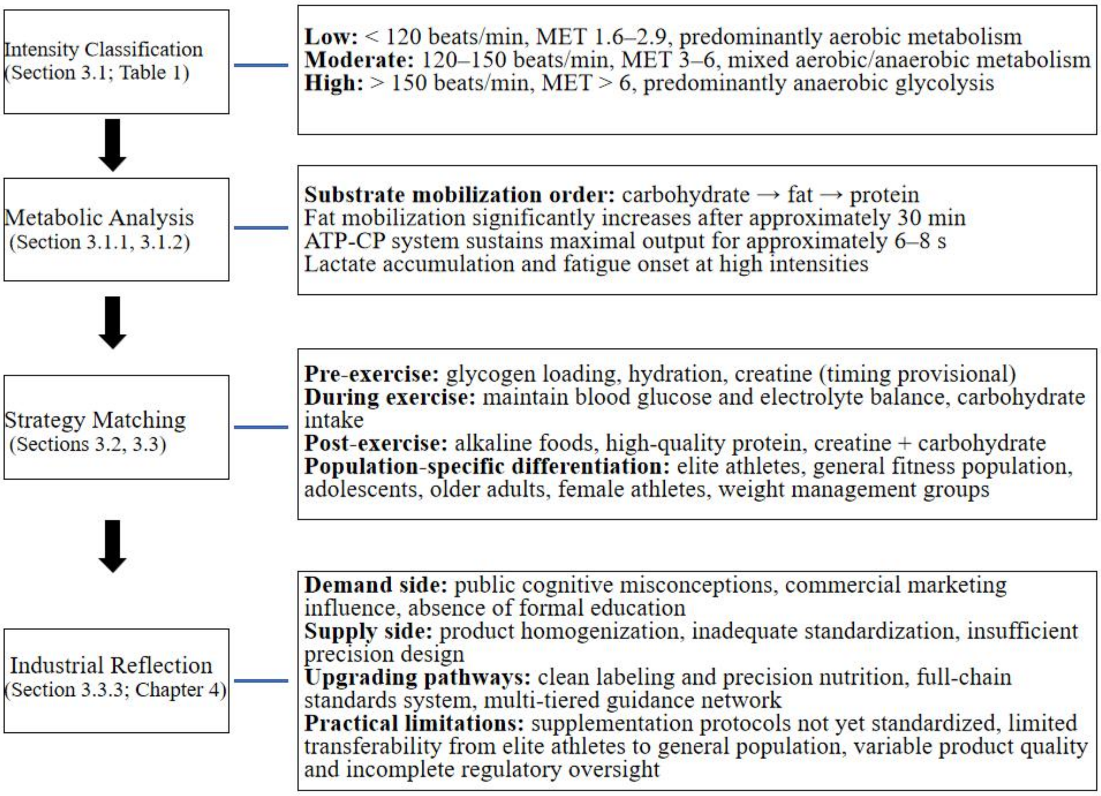

The framework consists of four components: (1) Intensity Classification, (2) Metabolic Analysis, (3) Strategy Matching, and (4) Industrial Reflection.
