# Supplementary material for: Cognition, Utilization and Industrial Development of Sports Nutrition Foods: An Evidence-Based Narrative Review
Source: Nutrients. 2026 Jun 13;18(12):1924. doi: 10.3390/nu18121924 (PMC13305693; doi:10.3390/nu18121924)
Supplement: Supplementary file 1 [file nutrients-18-01924-s001.zip › Supplementary_Table S1.pdf]

Table S1. Full search strategies for each database

Database	Search strategy	Search period	Records retrieved
CNKI	(SU % '运动营养食品' + '运动营养补剂' + '运动营养补充剂' + '运动饮料' + '蛋白补充剂' + '膳食补充剂' + 'sports nutrition food' + 'sports nutrition supplement' + 'sports nutrition product' + 'exercise nutrition food' + 'sports drink' + 'protein supplement' + 'dietary supplement') * (SU % '运动' + '体育' + '健身' + '锻炼' + '全民健身' + '运动强度' + '运动员' + '体力活动' + '训练' + 'exercise' + 'sport' + 'athlete' + 'fitness' + 'physical activity' + 'training' + 'national fitness' + 'exercise intensity') * (SU % '认知' + '知识' + '态度' + '感知' + '意识' + '行为' + '消费' + '使用' + '利用' + 'cognition' + 'knowledge' + 'attitude' + 'perception' + 'awareness' + 'behavior' + 'consumption' + 'utilization' + 'use') * (SU % '营养策略' + '补充策略' + '营养干预' + '食品开发' + '产品设计' + '产业发展' + '标准' + 'nutrition strategy' + 'supplementation strategy' + 'nutritional intervention' + 'food development' + 'product design' + 'industrial development' + 'standard')	2001-01-01 to 2025-03-31	40
Wanfang Data	主题:("运动营养食品" or "运动营养补剂" or "运动营养补充剂" or "运动饮料" or "蛋白补充剂" or "膳食补充剂") and 主题:("运动" or "体育" or "健身" or "锻炼" or "全民健身" or "运动强度" or "运动员" or "体力活动" or "训练") and 主题:("认知" or "知识" or "态度" or "感知" or "意识" or "行为" or "消费" or "使用" or "利用") and 主题:("营养策略" or "补充策略" or "营养干预" or "食品开发" or "产品设计" or "产业发展" or "标准")	2001-01-01 to 2025-03-31	21
PubMed	(("sports nutrition food"[Title/Abstract] OR "sports nutrition supplement"[Title/Abstract] OR "sports nutrition product"[Title/Abstract] OR "exercise nutrition food"[Title/Abstract] OR "sports drink"[Title/Abstract] OR "protein supplement"[Title/Abstract] OR "dietary supplement"[Title/Abstract]) AND ("exercise"[Title/Abstract] OR "sport"[Title/Abstract] OR "athlete"[Title/Abstract] OR "fitness"[Title/Abstract] OR "physical activity"[Title/Abstract] OR "training"[Title/Abstract] OR "national fitness"[Title/Abstract] OR "exercise intensity"[Title/Abstract]) AND ("cognition"[Title/Abstract] OR "knowledge"[Title/Abstract] OR "attitude"[Title/Abstract] OR "perception"[Title/Abstract] OR "awareness"[Title/Abstract] OR "behavior"[Title/Abstract] OR "consumption"[Title/Abstract] OR "utilization"[Title/Abstract] OR "use"[Title/Abstract]) AND ("nutrition strategy"[Title/Abstract] OR "supplementation strategy"[Title/Abstract] OR "nutritional intervention"[Title/Abstract] OR "food development"[Title/Abstract] OR "product design"[Title/Abstract] OR "industrial development"[Title/Abstract] OR "standard"[Title/Abstract])) AND ("2001"[Date - Publication] : "2025"[Date - Publication])	2001-01-01 to 2025-03-31	32

Database	Search strategy	Search period	Records retrieved
ScienceDirect	The search was conducted using the field code title-abs-key (title, abstract, keywords). Due to the platform-imposed limit of eight Boolean connectors per search field, the complete logic was split into three separate queries: (i) title-abs-key("sports nutrition food" OR "sports nutrition supplement" OR "sports drink" OR "protein supplement") AND title-abs-key("exercise" OR "sport" OR "athlete" OR "fitness") → 18 records; (ii) title-abs-key("sports nutrition food" OR "sports nutrition supplement" OR "sports drink") AND title-abs-key("cognition" OR "knowledge" OR "attitude" OR "behavior" OR "consumption") → 5 records; (iii) title-abs-key("sports nutrition food" OR "sports nutrition supplement" OR "sports drink") AND title-abs-key("nutrition strategy" OR "supplementation strategy" OR "nutritional intervention" OR "food development") → 2 records. After deduplication, 19 unique records were retained.	2001–2025	19 (after deduplication)

Note:

(1) Chinese search terms used in CNKI and Wanfang Data are as follows:运动营养食品 = sports nutrition food; 运动营养补剂 = sports nutrition supplement; 运动营养补充剂 = sports nutrition supplement; 运动饮料 = sports drink; 蛋白补充剂 = protein supplement; 膳食补充剂 = dietary supplement; 运动 = exercise; 体育 = sport; 健身 = fitness; 锻炼 = physical activity; 全民健身 = national fitness; 运动强度 = exercise intensity; 运动员 = athlete; 体力活动 = physical activity; 训练 = training; 认知 = cognition; 知识 = knowledge; 态度 = attitude; 感知 = perception; 意识 = awareness; 行为 = behavior; 消费 = consumption; 使用 = use; 利用 = utilization; 营养策略 = nutrition strategy; 补充策略 = supplementation strategy; 营养干预 = nutritional intervention; 食品开发 = food development; 产品设计 = product design; 产业发展 = industrial development; 标准 = standard. 主题 = Theme.

(2) Transparency note: The search strategies and record counts (CNKI: 40, without the Chinese–English expansion feature; Wanfang Data: 21, via manual combinations due to platform constraints; PubMed: 32; ScienceDirect: 19; totaling 112) reflect the original retrieval results at the manuscript preparation stage. When re-executed during revision, Wanfang Data yielded 72 records from an institutional network. This discrepancy does not affect the review's conclusions, as the majority of included articles were identified through backward citation tracking.
